# The Semi Centennial of the Discovery of Anæsthesia

**Published:** 1894-12

**Authors:** 


### The Semi-Centennial of the Discovery of Anæsthesia.

We call special attention to the following from the editorial pages of the Buffalo *Dental Practitioner and Advertiser:*

The year 1844 gave to surgery one of the greatest boons ever conferred upon suffering humanity. The discovery of anæsthesia marked a distinct era, and the credit of it, by universal consent, belongs to dentistry. As in the case of all other great discoveries, a number have claimed to originate it, but the consensus of opinion has awarded to the dentist, Horace Wells, of Hartford, Conn., the honor of first demonstrating it, and his name is enrolled among those highest in the temple of fame as being one of the greatest benefactors of any age. This is the semi-centennial year of its discovery, and dentistry should not miss the opportunity of reminding the world of the debt which it owes to a member of the young profession. Dentists themselves use anæsthetic agents largely in their practice, and they would be recreant to duty and prove themselves but ingrates if they did not in some way mark the occasion by a fitting observance of the anniversary.

The Odontological Society of Pennsylvania took action early in the year, by appointing a committee to take the matter into consideration. The Dental Society of the State of New York, at its annual meeting early in May, appointed a committee to take action upon the part of the dentists of the State. The Connecticut State Dental Society, at its annual meeting, passed resolutions and appointed a committee to arrange a semi-centennial celebration, and other societies will doubtless take similar action.

But the subject is too great and too comprehensive in interest to be monopolized by any single body of men. Every dentist throughout the world is concerned in the matter, and should have an opportunity in some way to assist in celebrating an event that must be to him a matter of personal pride. But the time is limited, and whatever is to be done must be done quickly. Half the year has flown without any general organization having been effected, and unless immediate action be taken the most momentous event in our professional history will not receive the attention which its importance demands.

